# Efficacy of exposure versus cognitive therapy in anxiety disorders: systematic review and meta-analysis

**DOI:** 10.1186/1471-244X-11-200

**Published:** 2011-12-20

**Authors:** Dennis Ougrin

**Affiliations:** 1 King's College London, Department of Child and Adolescent Psychiatry, Institute of Psychiatry PO85, De Crespigny Park, London, SE5 8AF, UK

## Abstract

**Background:**

There is growing evidence of the effectiveness of Cognitive Behavioural Therapy (CBT) for a wide range of psychological disorders. There is a continued controversy about whether challenging maladaptive thoughts rather than use of behavioural interventions alone is associated with the greatest efficacy. However little is known about the relative efficacy of various components of CBT. This review aims to compare the relative efficacy of Cognitive Therapy (CT) versus Exposure (E) for a range of anxiety disorders using the most clinically relevant outcome measures and estimating the summary relative efficacy by combining the studies in a meta-analysis.

**Methods:**

Psych INFO, MEDLINE and EMBASE were searched from the first available year to May 2010. All randomised controlled studies comparing the efficacy of exposure with cognitive therapy were included. Odds ratios (OR) or standardised means' differences (Hedges' g) for the most clinically relevant primary outcomes were calculated. Outcomes of the studies were grouped according to specific disorders and were combined in meta-analyses exploring short-term and long-term outcomes.

**Results:**

20 Randomised Controlled Trials with (n = 1,308) directly comparing the efficacy of CT and E in anxiety disorders were included in the meta-analysis. No statistically significant difference in the relative efficacy of CT and E was revealed in Post Traumatic Stress Disorder (PTSD), in Obsessive Compulsive Disorder (OCD) and in Panic Disorder (PD). There was a statistically significant difference favouring CT versus E in Social Phobia both in the short-term (Z = 3.72, p = 0.0002) and the long-term (Z = 3.28, p = 0.001) outcomes.

**Conclusions:**

On the basis of extant literature, there appears to be no evidence of differential efficacy between cognitive therapy and exposure in PD, PTSD and OCD and strong evidence of superior efficacy of cognitive therapy in social phobia

## Background

There is growing evidence of the effectiveness of Cognitive Behavioural Therapy for a wide range of psychological disorders [1].

There is a continued controversy about whether challenging maladaptive thoughts rather than use of behavioural interventions alone is associated with the greatest efficacy [2,3]. This issue appears central to any further enquiry into specific mediators of CBT effect.

Whether or not cognitive or behavioural interventions are more efficacious is also important from the public health point of view as behavioural techniques are considered to be generally simpler and cheaper both in therapists' training and in clinical applications [4]. The argument also has important implications regarding the theoretical role of cognitive factors in the aetiology and maintenance of psychiatric conditions.

Exposure and cognitive therapy are two most commonly used interventions in the treatment of anxiety disorders. Exposure has its roots in classical conditioning. In exposure patients make contact with the feared stimuli (either imaginary or in vivo) and this contact is maintained until the anxiety associated with the contact subsides. This process is termed habituation and it can only occur if the patients are prevented from using their usual escape or avoidance behaviour (extinction). Several versions of exposure exist. Systematic desensitisation (SD) is on the gentler part of the spectrum. It is based on the idea of reciprocal inhibition [5] proposing that two opposite emotions can not co-exist (e.g. fear and relaxation are mutually exclusive). In systematic desensitisation relaxation training is followed by gradual (usually imaginary) exposure to the feared stimuli starting with the least feared stimulus. In contrast, implosion entails an imaginary exposure to what would be the top of the hierarchy in SD, and flooding is the same procedure but done in vivo. Exposure has been described as the most effective way to treat fear [6] Research indicates that the efficacy of exposure is optimal when it is graduated, repeated and prolonged with practice tasks clearly specified [6].

For the purposes of this review the definition of cognitive therapy (CT) will be based on the work of David Clark [7] and could be summarised as follows: CT is the process of collaboration between the therapist and the patient leading to the identification of distorted thoughts (and/or maladaptive beliefs or assumptions) with the subsequent recognition, monitoring, logical analysis and empirical hypothesis-testing and finally the re-alignment of the patients' cognitions with reality. CT employs a number of techniques designed to re-align the distorted cognitions with reality, including thought monitoring and thought challenging, behavioural experiments, video feedback, surveys of opinion and the like. No matter what specific technique is employed, the framework of cognitive therapy remains constant: to identify the unhelpful cognition, to examine it in a collaborative way, to test its validity and then provide the patient with an opportunity to draw conclusions from their experience, often leading to revision of the original cognition.

### Cognitive Therapy and Exposure for Anxiety Disorders

Both CT and E alone or in combination have considerable efficacy for a range of anxiety disorders [8-11]. However little is known about the relative efficacy of various components of CBT. This review aims to compare the relative efficacy of CT and E for a range of anxiety disorders using the most clinically relevant outcome measures and estimating the summary relative efficacy by combining the studies in a meta-analysis.

## Methods

### Selection of studies

A literature search was carried out by using three electronic databases from the first available year to April 2010 without language limits. Psych INFO, MEDLINE and EMBASE were searched by using the following subject headings Cognitive Therapy OR Cognitive Behavior Therapy OR Behavior Therapy in combination with key words (* indicates truncation) component* OR process*, OR mediat* or moderat* or mechanis* OR exposure. These key words were chosen to capture the studies analysing therapeutic effects of different components of CBT. The studies were limited to treatment studies. Reference lists from the retrieved articles were also examined to find more relevant studies. In addition Anxiety, Post Traumatic Stress Disorder and Obsessive and Compulsive Disorder treatment guidelines were downloaded from the National Institute of Clinical Excellence (NICE)'s web site (http://www.nice.org.uk) and their reference lists were examined for relevant articles. Finally key investigators at the Oxford Cognitive Therapy Centre and at the Institute of Psychiatry (King's College London) Psychology Department were contacted in order to find out if there were any relevant unpublished studies. Authors were contacted if their data did not provide enough statistical information for the calculation of effect sizes.

The studies found were then downloaded into EndNote (version X2) and duplicates were deleted. The abstracts of the retrieved studies were reviewed and compared against the inclusion criteria. Where no definitive decision could be made on the basis of the abstract alone, the original paper was used.

Full text articles were assessed for eligibility by the author and by an independent senior researcher. Disagreements were resolved by consensus following a joint review of the articles and contacting the articles' authors if necessary.

### Inclusion and Exclusion Criteria

Only RCTs comparing exposure (E) and cognitive therapy (CT) in anxiety disorders were included whether individual or group treatment format was used. The studies of CT versus E+CT or E versus E+CT were excluded unless they contained pure CT versus E comparisons. The studies that contained an arm with pharmacological treatment were included provided (1) there was a comparison of E v CT; (2) there was no differential pharmacological treatment between the E and the CT groups.

Studies of CT with or without behavioural experiments and studies of exposure with or without relaxation training were included. However, studies of relaxation training without exposure were excluded.

Studies with more than 30% patient attrition were excluded. As other important study quality characteristics, allocation concealment and blindness were assessed and reported. Primary outcomes were included if they were relevant to the efficacy of E or CT for the condition treated, identified a priory, adequately reported and if their assessment was either adequately blinded or based on self-report. If no primary outcome measure was identified a priori, an outcome most closely linked to clinical efficacy was identified and the same quality considerations were applied as for the primary outcome measures. The outcomes were also included if they were possibly blinded but the actual use of blinding was uncertain. Outcomes that were definitely non-blinded present a high risk of bias and were excluded. Continuous outcomes had to be assessed by a standardized (preferably published) rating scale. They had to be assessed either as a self-report or by an independent (preferably blinded) rater. Ratings done by therapists were excluded as they were definitely not blinded and at serious risk of being biased. This is in accordance with the Cochrane Handbook [12].

### Individual study results

Both raw and transformed data were used for each outcome. Odds ratios (OR) were calculated for dichotomous and continuous outcomes and standardized means differences (Hedges' g) for continuous outcomes if different scales were used to report the same outcome. The effect size index Hedges' g is similar to Cohen's d (and can be interpreted similarly), but corrects for small-sample bias and is therefore more conservative in small samples. Effect estimates were calculated in such a way that a beneficial effect of CT was always represented by a negative effect size (for continuous outcomes) or by an odds ratio smaller than 1 (for dichotomous outcomes).

Pre-test means were assumed to be equal due to the randomisation.

### Combination of study results

Results for the same type of outcome (changes in symptoms) were combined across studies in a meta-analysis. Results of different outcomes were not combined. For outcomes where data were available from at least two studies, traditional meta-analytic summaries were calculated. Study results were pooled using a fixed effects model. When a substantial amount of statistical heterogeneity was found (when I^2 ^> 50% [12]) and could not be explained, a random effects model was used. RevMan 5, the programme used in Cochrane Collaboration's meta-analyses was utilised.

### Publication bias

Condition-specific funnel plots were constructed to examine the publication bias. No publication bias results in a funnel plot that is symmetrical around the mean effect size. The Trim and Fill method examines whether negative or positive trials are over- or underrepresented, accounting for the sample size. This information can then be used to recalculate the effect size estimate.

## Results

### Selection results

The initial search revealed a total of 1,612 articles. Having applied the exclusion criteria and having removed the duplicates this was reduced to 61 full text articles assessed for eligibility. None of the 61 articles were in a language other than English. There were disagreements about including 5 articles. Following a consensus meeting 20 RCTs were included in the final quantitative synthesis (additional file 1, PRISMA flowchart). The main reason for exclusion was unclear distinction between exposure and cognitive therapy. In particular the studies that did not have a clear focus on cognitive change were excluded from the CT group and the studies that did not have a clear focus on habituation were excluded from the E condition. The trials were grouped according to the condition studied: Obsessive Compulsive Disorder (OCD), Post-Traumatic Stress Disorder (PTSD), Panic Disorder with or without agoraphobia (PD) and Social Phobia (SP). Assessor blinding was adequate (or a self-reported tool was used as the main outcome measure) in 11 studies and uncertain in nine studies. Six countries were represented in the included studies: Canada, France, the UK, USA and the Netherlands. Together, the studies enrolled a total of N = 1,308 patients. Of those 1,044 were allocated to either CT or E condition and the rest were allocated to a comparison treatment or control conditions.

### General study characteristics

#### Studies of Cognitive Therapy versus Exposure in Obsessive Compulsive Disorder

Five studies have met the criteria for inclusion in this meta-analysis. Their results were reported in six articles [13-18] (Table S1, additional file 2). All included studies reported the pre- and post-treatment values for Yale-Brown Obsessive Compulsive Scale (Y-BOCS). This is a semi-structured clinician-administered interview providing a score (range 0-40) for both obsessions (range 0-20) and compulsions (range 0-20) along five dimensions: time spent, interference, distress, resistance, and control [19,20]. One of the studies [14] contained a condition of E + fluvoxamine and CT + fluvoxamine. These two conditions were excluded due to drop out rates exceeding 30% (36% and 41% respectively); however the pure E and CT groups were included in the review and the meta-analysis. The studies comparing Rational Emotive Therapy (RET) with Exposure [21,22] were excluded as the RET model lacks theoretical coherence and empirical evidence afforded by the modern cognitive behaviour models.

#### Studies of Cognitive Therapy versus Exposure in Post Traumatic Stress Disorder

Five studies met the criteria for inclusion in this meta-analysis [23-27] (Table S2, additional file 3). Three of the included studies reported the pre- and post-treatment values for Clinician-Administered PTSD scale (CAPS) [28]. CAPS has 30 items, assessor-rated, rating frequency and intensity of the 17 PTSD symptoms and 8 associated features of PTSD (built over acts of commission or omission, survivor guilt, homicidality, disillusionment with authority, hopelessness, memory impairment and forgetfulness, sadness and depression and feeling overwhelmed). Each item is rated 0-4 for intensity and frequency. One study [26] used an aggregate PTSD severity index calculated by adding the interviewer's severity rating of the following PTSD symptoms: reliving experiences, nightmares, flashbacks, avoidance of reminders and thoughts of the assault, impaired leisure activities (e.g., reduced socializing), sense of detachment, blunted affect, disturbed sleep, memory and concentration difficulties, hyperalertness, increased startle response, feelings of guilt, and increased fearfulness. One study used PTSD Symptom Scale - Interview (PSS-I) [29] The scale contains 17 items that diagnose PTSD according to Diagnostic and Statistical Manual of Mental Disorders-III-Revised (DSM-III-R) criteria and assess the severity of PTSD symptoms.

#### Studies of Cognitive Therapy versus Exposure in Panic Disorder with or without Agoraphobia

Seven studies met the criteria for inclusion in this meta-analysis [30-35] (Table S3, additional file 4). Six included studies reported the pre- and post-treatment values for the proportion of the patients free from panic attacks based on self-report. One study [33] only gave the raw number of reported panic attacks; however the proportion of the patients free from panic attack was calculated on the basis of the data provided.

#### Studies of Cognitive Therapy versus Exposure in Social Phobia

Three studies met the inclusion criteria for this meta-analysis [36-38] (Table S4, additional file 5). Two studies used a social phobia composite score as the main outcome measure and one study used Social Phobia and Anxiety Inventory (SPAI) as the main outcome measure. The SPAI [39] is a 109-item self-report instrument that has been widely used to assess the cognitive, somatic and behavioural dimensions of social phobia. One of the studies used a comparison between Cognitive Therapy and Exposure with Fluoxetine or Exposure with Placebo. The arm including Fluoxetine treatment was excluded as per inclusion criteria.

## Meta-analyses

### Meta-analysis of Cognitive Therapy versus Exposure Efficacy in Obsessive Compulsive Disorder

#### Short-term outcomes

**Figure 1:**
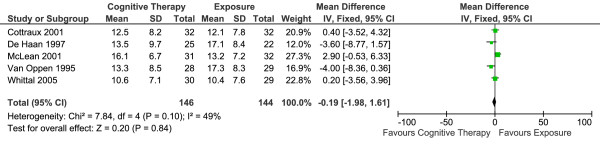
**Cognitive therapy versus exposure for OCD**. Meta-analysis: short-term outcomes Note. SD = Standard Deviation; IV = Inverse Variance; OCD = Obsessive Compulsive Disorder.

Five studies reported short-term efficacy (the end of treatment mean Y-BOCS score) of CT versus E in 290 patients (Table 1). The overall effect is summarised in Figure 1. There was no statistically significant difference between the two conditions. Fixed-effects model was used to estimate the overall effect as there was no evidence of significant heterogeneity (I^2 ^= 49%).

**Table 1:** Results of the studies of exposure versus cognitive therapy in OCD, Y-BOCS total score

Study	Main OM	Pre-treatment		End of Treatment		Longest follow up	Longest follow up	
				
		CT	E	CT	E	(weeks)	CT	E
Van Balkom 1998	Y-BOCS	25.0(6.6)	24.7 (7.7)	13.5 (9.7)	17.1 (8.4)	26	11.0 (7.5)	11.1 (7.1)

Van Oppen 1995	Y-BOCS	24.1 (5.5)	25.4 (7.0)	13.3 (8.5)	17.3 (8.3)	NA	NA	NA

Cottraux 2001	Y-BOCS	28.6 (5.1)	28.5 (4.9)	12.5 (8.2)	12.1 (7.8)	52	13.6 (8.8)	17.4 (8.5)

McLean 2001	Y-BOCS	21.9 (5.8)	21.8 (4.6)	16.1 (6.7)	13.2 (7.2)	104	17.3 (8.4)	12.8 (7.2)

Whittal 2005	Y-BOCS	23.50 (4.3)	21.66 (5.9)	10.6 (7.1)	10.4 (7.6)	104	10.3 (6.2)	11.2 (8.8)

Note. CT = Cognitive Therapy; E = Exposure; Y-BOCS = Yale-Brown Obsessive Compulsive Scale

#### Long-term outcomes

**Figure 2:**
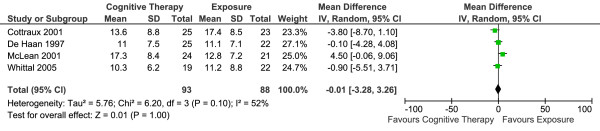
**Cognitive therapy versus exposure for OCD**. Meta-analysis: long-term outcomes Note. SD = Standard Deviation; IV = Inverse Variance; OCD = Obsessive Compulsive Disorder.

Four studies reported long-term efficacy (the mean Y-BOCS score at the point of the longest reported follow up) of CT versus E in 181 patients (Table 1). The overall effect is summarised in Figure 2. There was no statistically significant difference between the two conditions. Random-effects model was used to estimate the overall effect as there was evidence of significant heterogeneity (I^2 ^= 51%).

### Meta-analysis of Cognitive Therapy versus Exposure Efficacy in Post Traumatic Stress Disorder

#### Short-term outcomes

**Figure 3:**
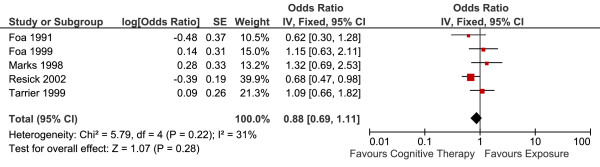
**Cognitive therapy versus exposure for PTSD**. Meta-analysis: short-term outcomes Note. SD = Standard Deviation; IV = Inverse Variance; PTSD = Post Traumatic Stress Disorder.

Five studies reported short-term efficacy of CT versus E in 287 patients (Table 2). The overall effect (the end-of-treatment standardized mean differences, Hedge's g) is summarised in Figure 3. There was no statistically significant difference between the two conditions. Fixed-effects model was used to estimate the overall effect as there was no evidence of significant heterogeneity (I^2 ^= 29%).

**Table 2:** Results of the studies of exposure versus cognitive therapy in PTSD, mean PTSD rating scales' scores

Study	Main OM	Pre-treatment		End of Treatment		Longest follow up	Longest follow up	
				
		CT	E	CT	E	(weeks)	CT	E
Terrier 1999	CAPS	76.93 (15.4)	71.76 (19.59)	50.82 (23.99)	48.24 (30.25)	52	52.48 (24.09)	45.16 (28.26)

Marks 1998 (reported in Lovell et al 2001)	CAPS	74.7 (18.1)	61.9 (15.7)	39.5 (23.7)	31.5 (30.7)	26	29.4 (18.7)	14.3 (16.2)

Resick 2008	CAPS	73.87 (21.04)	70.38 18.65	31.32 (37.00)	44.76 31.55	26	31.03 (27.57)	35.90 (27.09)

Foa 1991	PTSD severity	24.3 (6.1)	25.01 (4.64)	9.89 (4.2)	13.56 (10)	12	12.33 (9.59)	10.44 (8.22)

Foa 1999	PSS-I	29.42 (8.69)	29.48 (9.94)	12.89 (8.96)	11.7 (7.32)	52	12.64 (14.71)	10.69 (8.96)

Note. CT = Cognitive Therapy; E = Exposure; CAPS = Clinician Administered PTSD scale; PSS-I = PTSD Symptom Scale - Interview

#### Long-term outcomes

**Figure 4:**
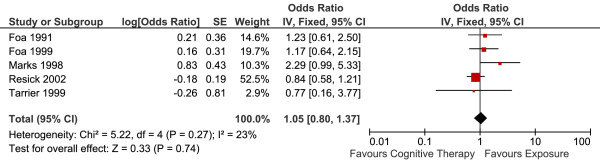
**Cognitive therapy versus exposure for PTSD**. Meta-analysis: long-term outcomes Note. SD = Standard Deviation; IV = Inverse Variance; PTSD = Post Traumatic Stress Disorder.

Four studies reported long-term efficacy reported long-term efficacy of CT versus E in 226 patients (Table 2). The overall effect (the standardized means differences, Hedge's g at the longest-reported follow up) is summarised in Figure 4. There was no statistically significant difference between the two conditions. Fixed-effects model was used to estimate the overall effect as there was no evidence of significant heterogeneity (I^2 ^= 0).

### Meta-analysis of Cognitive Therapy versus Exposure Efficacy in Panic Disorder with or without Agoraphobia

#### Short-term outcomes

**Figure 5:**
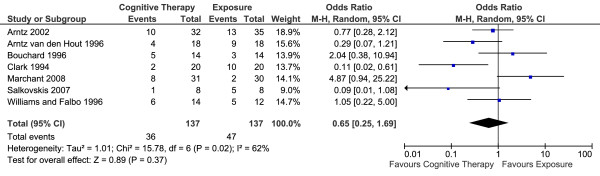
**Cognitive therapy versus exposure for panic disorder**. Meta-analysis: short-term outcomes Note. SD = Standard Deviation; IV = Inverse Variance.

Seven studies reported short-term efficacy (the end of treatment proportion of panic-free patients) of CT versus E in 274 patients (Table 3). The overall effect is summarised in Figure 5. There was no statistically significant difference between the two conditions. Random-effects model was used to estimate the overall effect as there was evidence of significant heterogeneity (I^2 ^= 68%).

**Table 3:** Results of the studies of exposure versus cognitive therapy in panic disorder, panic-free patients

Study	Main OM	Pre-treatment		End of Treatment		Longest follow up	Longest follow up	
				
		CT	E	CT	E	(weeks)	CT	E
Arntz 2002	Panic free	0	0	69%	63%	26	75%	71%

Bouchard 1996	Panic free	0	0	64%	79%	26	43%	71%

Marchant 2008	Panic free	0	0	79%	90%	52	59%	82%

Salkovskis 2007	Panic free	0	0	63%	13%	NA	NA	NA

Williams and Falbo 1996	Panic free	0	0	57%	58%	104	50%	80%

Clark 1994	Panic free	0	0	90%	50%	64	85%	47%

Arntz and van den Hout 1995	Panic free	0	0	78%	50%	26	78%	50 %

Note. CT = Cognitive Therapy; E = Exposure

#### Long-term outcomes

**Figure 6:**
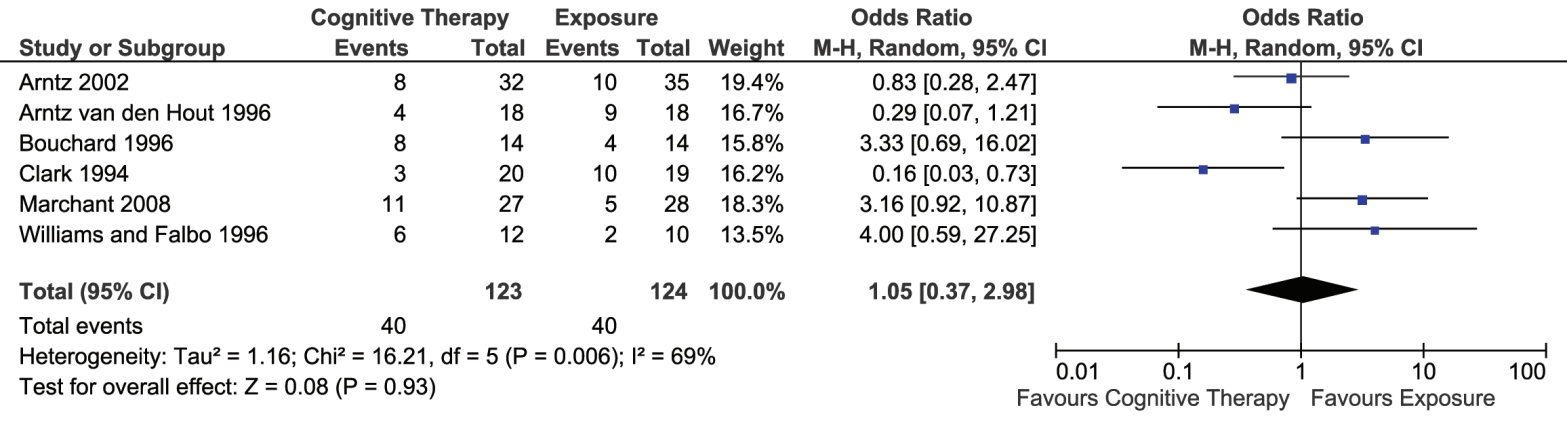
**Cognitive therapy versus exposure for panic disorder**. Meta-analysis: long-term outcomes Note. SD = Standard Deviation; IV = Inverse Variance.

Six studies reported long-term efficacy (the end of treatment proportion of panic-free patients) of CT versus E in 247 patients (Table 3). The overall effect is summarised in Figure 6. There was no statistically significant difference between the two conditions. Fixed-effects model was used to estimate the overall effect as there was no evidence of significant heterogeneity (I^2 ^= 24%).

### Meta-analysis of Cognitive Therapy Efficacy versus Exposure in Social Phobia

#### Short-term outcomes

**Figure 7:**
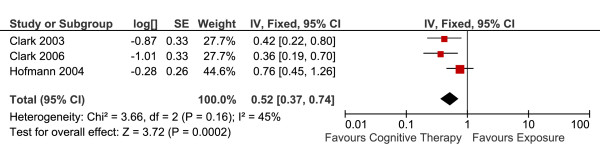
**Cognitive therapy versus exposure for social phobia**. Meta-analysis: short-term outcomes Note. SD = Standard Deviation; IV = Inverse Variance.

Three studies reported short-term efficacy of CT versus E in 128 patients (Table 4). The overall effect (the end-of-treatment standardised means differences, Hedge's g) is summarised in Figure 7. There was a statistically significant difference favouring CT versus E. Fixed-effects model was used to estimate the overall effect as there was no evidence of significant heterogeneity (I^2 ^= 45%).

**Table 4:** Results of the studies of exposure versus cognitive therapy in social phobia, effect size

Study	Effect size	End of Treatment		Longest follow	Longest follow up up (weeks)	
				
		CT v E (SE)	95% CI	(weeks)	CT v E (SE)	95% CI
Clark 2003	Hedge's g	-0.87 (0.33)	(-1.52) - (-0.22)	52	NA	NA

Clark 2006	Hedge's g	-1.01 (0.33)	(-1.65) - (-0.36)	52	-0.85 (0.32)	(-1.48) - (-0.22)

Hofmann 2004	Hedge's g	-0.28 (0.26)	(-0.79) - 0.23	26	-0.7 (0.34)	(-1.37) - (-0.03)

Note CT = Cognitive Therapy; E = Exposure

#### Long-term outcomes

**Figure 8:**
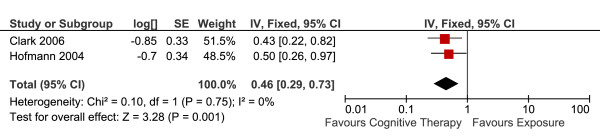
**Cognitive therapy versus exposure for social phobia**. Meta-analysis: long-term outcomes Note. SD = Standard Deviation; IV = Inverse Variance.

Two studies totalling 75 patients reported long-term efficacy (the standardised means differences, Hedge's g, at the longest-reported follow up) of CT versus E (Table 4). The overall effect is summarised in Figure 8. There was a statistically significant difference favouring CT versus E. Fixed-effects model was used to estimate the overall effect as there was no evidence of significant heterogeneity (I^2 ^= 0)

#### Publication bias

Condition-specific funnel plots did not indicate publication bias. Small number of studies in each comparison, however, means that the assessment of publication bias needs to be interpreted with caution.

#### Other anxiety disorders

There are studies examining relative efficacy of exposure and cognitive therapy in other anxiety disorders: hypochondriasis [40] and generalised anxiety disorder (GAD) [41]. More studies are needed before a summary effect of CT versus E could be estimated in these conditions.

## Discussion

### Cognitive Therapy v Exposure in OCD PTSD and PD

This meta-analysis provided no evidence of a statistically significant difference in efficacy between exposure and cognitive therapy for the clinically relevant outcome measures across RCTs studying OCD, PTSD, and panic disorder with or without agoraphobia.

This does not mean that cognitive changes do not mediate the treatment response as it is possible that cognitions could change and mediate treatment through means other than cognitive therapy. Cognition changes in the trials of pharmacotherapy have indeed been demonstrated [42]. Behavioural work could also result in cognitive changes even in the absence of direct cognitive therapy [43]. There is also growing evidence of the role cognitive mediation might play in achieving clinical improvement during exposure therapy [44].

Equally, it is possible that exposure plays an important role in some cognitive interventions, behavioural experiments in particular.

In addition, it is possible that the different components of CBT examined in this study may work on different systems, such that producing change in one system (for example, the behavioural system) will produce changes in other systems (such as the cognitive system) [41,45]. If this was the case then it is not surprising that component analysis studies show similar outcomes for exposure and cognitive therapy in many conditions.

### Cognitive Therapy v Exposure in Social Phobia

CT was found to be superior to E in both short term and long-term outcomes in Social Phobia (effect size differences from 0.28 to 1.01). A re-evaluation of the data using a random effects analysis did not change the overall conclusion. This differential efficacy has not been shown previously and is potentially of significant clinical importance. The apparent superiority of CT versus E in social phobia needs to be accepted cautiously. First, the studies included in this meta-analysis were conducted by two research groups that may be especially proficient in the use of cognitive therapy. Second, E condition in two of the three studies was individual, whereas it may be more effective in group setting. Having said this, E condition in all three studies demonstrated significant effect size (0.56- 1.46) and in one study was based on a manual developed by the investigators and tested in previous studies [38].

It may be that the nature of social phobia makes cognitive interventions especially important in this condition. The effect of CT may be mediated by an early change in estimated social cost [38] partly explaining the long-term CT superiority over E. In addition, there are studies demonstrating that behavioural experiments may be more effective in anxiety reduction than exposure alone [46] particularly when an external focus of attention is used for confronting social anxiety-provoking situations. Exposure in social phobia may be of limited value without some skills-based behavioural response. It is also possible that the duration of exposure in social phobic situations is not sufficient for it to be effective as many social encounters have brief duration. Cognitive therapy draws on a range of techniques to achieve therapeutic change, some of which have already been demonstrated to be superior to exposure alone. For instance, the impact of video feedback, imagery work and the negative consequences of self-directed attention have all been demonstrated experimentally [47,48]. In summary, CT appears to be more effective than exposure alone in social phobia; however more studies from a range of research groups are needed to confirm this finding.

### Is there a need to challenge thoughts in CBT?

Since there is no evidence of differential efficacy of CT and E for some conditions, is it therefore necessary to employ cognitive interventions in the treatment of anxiety disorders? The arguments in favour of this proposition could be summarised in this way. 1. There is some evidence that cognitive interventions and behavioural interventions have similar effect sizes for certain conditions [10] or there is even a superiority of modified behavioural interventions compared with purely cognitive interventions [49]. 2. The rapid response argument [50] centres on the idea that many patients show an improvement early in the course of CBT [51] making the effect of cognitive interventions an unlikely mediator of this change (on the assumptions that cognitive interventions require several weeks to be implemented fully) 3. The mediation argument proposes that there is no compelling evidence demonstrating that cognitive change is the underlying mechanism of improvement in CBT. 4. The cost-effectiveness argument where behavioural interventions are seen as cheaper to teach and to implement.

This meta-analysis contributed to the discussion on the first of these points. It would appear that even within the field of anxiety disorders there is no categorical evidence of equivalence between cognitive and behavioural interventions and it may be that cognitive and behavioural interventions may be more suitable for certain conditions. Having said this there was no evidence of a difference in effect size between CT and E in three out of four anxiety conditions.

The rapid response argument would appear to be the weakest of the four. There is plenty of evidence documenting cognitive changes early in the cognitive treatment [33,52]. In addition, there is more and more evidence of cognitive change not being specific to cognitive therapy [42].

The mediation argument is the most complex one as the criteria for mediation differs depending on the study design [53,54]. For RCTs the test of mediation is proposed to include these variables: (1) the proposed mediator correlates with treatment assignment; (2) the mediator has either a main or interactive effect on outcome; and (3) changes in the mediator variable precede changes in the dependent variable. Very few studies address all of these criteria. In addition the issue is confounded by the rapid response phenomenon. The literature on cognitive change as a mediator of improvement is growing, although it remains somewhat inconsistent [38,42,55]. Future studies are likely to clarify the mechanisms of change in CBT further.

The cost-effectiveness argument is the least well studied. There is some evidence of a greater cost of teaching a range of cognitive skills as opposed to exposure alone. The cost of implementation of exposure versus cognitive therapy is also poorly studied but is likely to be lower. It is worth mentioning that all but one of the studies included in this meta-analysis allocated equal time to both CT and E treatment. The studies of relative cost-effectiveness of E versus CT are timely.

### Limitations

The results of a meta-analysis depend firstly on the results of the individual studies included. Therefore, their limitations should be mentioned here first. The studies with very high risk of bias (e.g. with very high drop-out rates) were excluded from the review. Although only RCTs were included, some would appear to have had 'weaker' designs than the strongest ones that exist. This was done to enhance external validity. In half of the studies it was uncertain whether or not outcome assessment was adequately blinded. Similarly, concealment of allocation was often uncertain. In addition no standardised quality assessment tools were used to assess the quality of the studies included. One significant limitation of most studies included is the lack of an explicitly designated a-priori primary outcome measure. None-the-less the outcome measures included in the meta-analysis were either designated as primary or have been well validated in other studies.

Although great care was taken to include pure cognitive therapy versus pure exposure approach to treatment it is possible that a certain amount of E and CT were used across the comparison studies. In addition the effect of adding CT to E (or E to CR) was not studied in this meta-analysis. Based on that, a logical and useful next step in this field of research would be to examine the additive value of E and CT across different conditions.

Only the principal clinical efficacy outcomes were examined in this meta-analysis. On one hand this allowed for a comprehensive and straightforward comparison of two commonly used techniques. On the other hand, it may be that comparing the outcomes other than clinical efficacy (for example depressive symptomatology, drop out rates or quality of life measures) could have contributed to a different interpretation of the results.

The potential impact of researcher allegiance has been much debated in psychotherapy research [56]. Although it was not the focus of this review, it should be mentioned here as a potential limitation. It is plausible to assume that most studies in this review were carried out by researchers who have positive allegiances to either cognitive or behavioural interventions. However, the high consistency of the efficacy identified in this review makes bias from researcher allegiance unlikely.

Finally, the small number of studies with small sample size deserves mentioning as a limitation coupled with selected demographic characteristics of the subjects (primarily Caucasian adults) and the treatments delivered mainly by highly trained therapists in the centres of excellence. These factors reduce the generalisability of the findings of this meta-analysis and limit the potential for sensitivity analyses on possible confounding variables or predictor variables.

### Implications for Practice

The findings of this review have several implications for practice. First, they underline the value of CBT as an effective treatment in mental health care. Second, the findings imply that there is no evidence of statistically significant difference in E versus CT across several but not all conditions. It has to be noted, however, that the extent of individual benefit from E versus CT might vary from patient to patient. Some may respond better to one of the two interventions and some might do better with a combination.

The lack of difference of efficacy between E and CT in OCD, PTSD and PD is important for clinical reasons. The British NICE guidelines state that ERP must be offered as part of CBT for OCD. The guideline development group conducted several comparisons, however only one study was used to compare the efficacy of CT v ERP [57]. This study showed no significant difference in the initial analysis of the efficacy of CT v ERP (these findings were used for the present study); however after adjustment for medication use a marginally significant result was reported in favour of ERP (p = 0.049). Further studies are required to inform the NICE recommended interventions for OCD

The NICE guidelines for PTSD recommend the use of trauma-focused CBT or EMDR as a first line psychological treatment. Equal efficacy of CT versus E reported in this meta-analysis is in line with this recommendation.

The NICE guidelines on the management of Panic Disorder also recommend CBT as the first line psychological treatment with no specific recommendation of exposure or cognitive therapy as an important ingredient. The comparisons between cognitive and behavioural interventions used to inform the guideline development was based on two studies [30,58] one of which was excluded from this meta-analysis due to the main outcome measure not being reported [58].

At present there are no NICE guidelines on the management of social phobia. Clinicians might like to note the relative superior efficacy of cognitive therapy over exposure found in this meta-analysis.

A further implication for practice, the lack of evidence for a difference of efficacy between E and CT in PD, OCD and PTSD raises the question of differential indication for behavioural versus cognitive treatment. If E v. CT is not the main determinant of the effect of CBT, then what other criteria might be more fruitful in determining who should receive a particular CBT intervention? Psychotherapy researchers have argued that factors such as the match between therapist and client and the client's motivation for a specific type of therapy should be recognised more [59]. The use of such "soft" indications can often be more fruitful than an uncritical 'prescription' based on the availability or the services and the therapists' allegiance. For policy makers it will be important to take into account the findings of this review to consider the relative need of training in E v. CT.

There are no good studies documenting the economic implications of teaching and supervising CT v. E and it may be important to undertake such studies especially bearing in mind the recent government initiatives promoting wider use of CBT in the UK and elsewhere.

## Conclusions

This systematic review and meta-analysis compared the clinical efficacy of exposure and cognitive therapy in the treatment of anxiety disorders. On the basis of the extant literature, there appears to be no evidence of differential efficacy between exposure and cognitive therapy in PD, PTSD and OCD and strong evidence of superior efficacy of cognitive therapy in social phobia. In light of the findings of this meta-analysis the necessity of including ERP in the treatment of OCD appears to be questionable. Cognitive interventions might have a particularly important role to play in social phobia. This finding should be noted when the clinical guidelines for social phobia are developed.

## Competing interests

The authors declare that they have no competing interests.

## Pre-publication history

The pre-publication history for this paper can be accessed here:

http://www.biomedcentral.com/1471-244X/11/200/prepub

## Supplementary Material

Additional file 1 **PRISMA flow diagram**. Note. PRISMA = Preferred Reporting Items for Systematic Reviews and Meta-Analyses. Click here for file

Additional file 2 **Table S1 Studies of cognitive therapy versus exposure in obsessive compulsive disorder**. Legend: Note. CT = Cognitive Therapy; E = Exposure; ITT = Intention to Treat; OCD = Obsessive Compulsive Disorder; M = Mean. Click here for file

Additional file 3 **Table S2 Studies of cognitive therapy versus exposure in post traumatic stress disorder**. Note. CT = Cognitive Therapy; E = Exposure; ITT = Intention to Treat; PTSD = Post Traumatic Stress Disorder; M = Mean. Click here for file

Additional file 4 **Table S3 Studies of cognitive therapy versus exposure in panic disorder with or without agoraphobia**. Note. CT = Cognitive Therapy; E = Exposure; ITT = Intention to Treat; PD = Panic Disorder; M = Mean. Click here for file

Additional file 5 **Table S4 Studies of cognitive therapy versus exposure in social phobia**. Note. CT = Cognitive Therapy; E = Exposure; ITT = Intention to Treat. Click here for file
