# Supplementary material for: Efficacy of exposure versus cognitive therapy in anxiety disorders: systematic review and meta-analysis
Source: BMC Psychiatry. 2011 Dec 20;11:200. doi: 10.1186/1471-244X-11-200 (PMC3347982; doi:10.1186/1471-244X-11-200)
Supplement: Additional file 1 — PRISMA flow diagram. Note. PRISMA = Preferred Reporting Items for Systematic Reviews and Meta-Analyses. [file 1471-244X-11-200-S1.DOC]

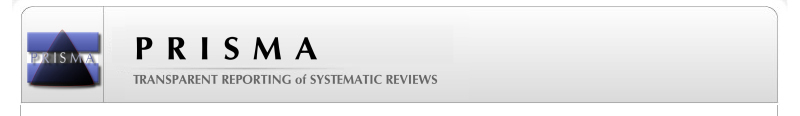
**PRISMA 2009 Flow Diagram**

**Screening**

**Included**

**Eligibility**

**Identification**

Records identified through database searching
(n = 1,612 )

Additional records identified through other sources
(n = 24 )

Records after duplicates removed
(n = 933 )

Records screened
(n = 933 )

Records excluded
(n = 872 )

Full-text articles assessed for eligibility
(n = 61 )

Full-text articles excluded, with reasons
(n = 41; 37 due to lack of distinction between exposure and cognitive therapy; 2 due to absence of exposure in the behavioural arm and 2 due to not meeting the quality criteria)

Studies included in qualitative synthesis
(n = 22 )

Studies included in quantitative synthesis (meta-analysis)
(n = 20 )
