# Supplementary material for: Efficacy of exposure versus cognitive therapy in anxiety disorders: systematic review and meta-analysis
Source: BMC Psychiatry. 2011 Dec 20;11:200. doi: 10.1186/1471-244X-11-200 (PMC3347982; doi:10.1186/1471-244X-11-200)
Supplement: Additional file 2 — Table S1 Studies of cognitive therapy versus exposure in obsessive compulsive disorder. Legend: Note. CT = Cognitive Therapy; E = Exposure; ITT = Intention to Treat; OCD = Obsessive Compulsive Disorder; M = Mean. [file 1471-244X-11-200-S2.DOC]

**Table S1 Studies of Cognitive Therapy versus Exposure in Obsessive Compulsive Disorder**

| **Study** | **Design and study quality** | **Treatment/ follow up (weeks)** | **Participants** | | | | | | | | | |
| --- | --- | --- | --- | --- | --- | --- | --- | --- | --- | --- | --- | --- |
|  |  |  | **Clinical condition and setting** | **ITT** | | **Demographic** | | | **Interventions** | | **Comparison** | **Outcome** **measures** |
|  | | | | | | | | | **Type** | **No. of sessions** |  |  |
| Van Balkom 1998 | Design: parallel allocation; concealment: unknown blindness: not reported;  attrition: 29% | Treatment:16 follow up: 26 | Diagnosis: OCD  Setting: tertiary OCD specialist centres outpatients  Country: The Netherlands | Completers and ITT. ITT outcomes not specified | | *N* = 47 Age: 18-65 (*M* = 35) Sex: 42% male | | | CT (n=25)  E (n=22) | 16 | (1) CT + fluvoxamine  (2) E + fluvoxamine | 1. Yale-Brown Obsessive-Compulsive Scale  2. Symptom Checklist -90  3. Beck Depression Inventory |
| Cottraux 2001 | Design: parallel allocation; concealment: unknown; blindness: assessors blind to treatment allocation;  attrition: 20% | Treatment 16; follow up: 52 | Diagnosis: OCD  Setting: university hospital outpatients  Country: France | Completers and ITT | | *N* = 65 Age: 18-65 (*M* = 36) Sex: 25% male | | | CT (n=32)  E (n=33) | 20 | None | 1. Yale-Brown Obsessive-Compulsive Scale  2. NIMH-Obsessive Compulsive Scale  3. Quality of Life Scale  4. Beck Depression Inventory  5. Salkovskis Responsibility Scale  6. Behavioural Avoidance Test  7. Beck Depression Inventory  8. The Intrusive  Thoughts and Their Interpretation Questionnaire  9. The Obsessive Thoughts Checklist  10. Marks’ Fear Questionnaire |
| McLean 2001 | Design: parallel allocation; concealment: unknown; blindness: unknown;  attrition: 17% | Treatment: 12; follow up: 104 (reported in Whittal 2008) | Diagnosis: OCD  Setting: outpatients, group treatment  Country: Canada | Completers and partial ITT (last observation carried forward) | | | *N* = 76 Age: 18-56 (*M* = 35) Sex: 25% male | | Group CT (n=34)  Group E (n=42) | 12 | Wait list | 1. Yale-Brown Obsessive-Compulsive Scale  2. Beck Depression Inventory  3. Responsibility Attitude Scale  4. Beck Depression Inventory  5. Thought Action Fusion Scale  6. Inventory of Beliefs related to Obsessions |
| Whittal 2005 | Design: parallel allocation; concealment: unknown; blindness: unknown;  attrition: 21% | Treatment: 12; follow up: 104 (reported in Whittal 2008) | Diagnosis: OCD  Setting: outpatients  Country: Canada | Completers and partial ITT (last observation carried forward) | N=71 Age: 18-65 (*M* = 35) Sex: 37% male | | | CT (n=34)  E (n=37) | | 12 | None | 1. Yale-Brown Obsessive-Compulsive Scale  2. Obsessional Beliefs Scale  3. Beck Depression Inventory  4. Interpretations of Intrusions Inventory |

Note. CT = Cognitive Therapy; E = Exposure; ITT = Intention to Treat; OCD = Obsessive Compulsive Disorder; M = Mean.
