# Supplementary material for: Efficacy of exposure versus cognitive therapy in anxiety disorders: systematic review and meta-analysis
Source: BMC Psychiatry. 2011 Dec 20;11:200. doi: 10.1186/1471-244X-11-200 (PMC3347982; doi:10.1186/1471-244X-11-200)
Supplement: Additional file 3 — Table S2 Studies of cognitive therapy versus exposure in post traumatic stress disorder. Note. CT = Cognitive Therapy; E = Exposure; ITT = Intention to Treat; PTSD = Post Traumatic Stress Disorder; M = Mean. [file 1471-244X-11-200-S3.DOC]

**Table 2 Studies of Cognitive Therapy versus Exposure in Post Traumatic Stress Disorder**

| **Study** | **Design and study quality** | **Treatment/ follow up (weeks)** | **Participants** | | | | | | |
| --- | --- | --- | --- | --- | --- | --- | --- | --- | --- |
|  |  |  | **Clinical condition and setting** | **ITT** | **Demographic** | **Interventions** | | **Comparison** | **Outcome scales** |
|  | | | | | | **Type** | **No. of sessions** |  |  |
| Tarrier 1999 | Design: parallel allocation; concealment: unknown; blindness: assessors unaware of treatment condition;  attrition: 25% | Treatment: 24; follow up: 52 | Diagnosis: PTSD  Setting: University clinic, outpatients  Country: UK | Completers | N=72 Age-adults (*M* = 38) Sex: 60% male | CT (n=37)  E (n=35) | 16 | None | 1. Beck Anxiety Inventory  2. Beck  Depression Inventory  3. Impact of Events Scale  4. Clinician Administered PTSD scale  5. General Health Questionnaire -28 |
| Marks 1998 | Design: parallel allocation; concealment: unknown; blindness: assessors unaware of treatment condition; Attrition: 27% | Treatment: 16; follow up: 36 | Diagnosis: PTSD  Setting: tertiary centre, out-patients  Country: UK | Completers | N=42 Age – adults (*M* = 38) Sex: 65% male | CT (n=19)  E (n=23) | 10 | CT+E;  Relaxation | 1.Global Improvement  2. General Health Questionnaire - 28  3.Fear questionnaire  4. Beck  Depression Inventory  5. Impact of Events Scale  6. Clinician Administered PTSD scale  7. Main problem  8. Work/social adjustment  9. Total of 4 goals |
| Resick 2008 | Design: parallel allocation; concealment: unknown; blindness: assessors unaware of treatment condition; attrition: 18% | Treatment 12; follow up: 36 | Diagnosis: PTSD  Setting: tertiary centre, out-patients  Country: USA | Partial ITT (n=150): last observation carried forward | N=162 age-adults (M=35.4) all female | CT (n=56)  E (n=55) | CT= 12  E=7 (*)  *matched for time=12 hours | CT+E (n=51) | 1. Trauma Related Guilt Inventory  2. Beck  Depression Inventory  3. Experience of Shame Scale  4. Clinician Administered PTSD scale  5.Personal Beliefs and Reactions Scale  6. Posttraumatic Diagnostic Scale  7. State-Trait Anger Expression Inventory  8. State-Trait Anxiety Inventory |
| Foa 1991 | Design: parallel allocation; concealment: unknown; blindness: unknown; attrition: 15% | Treatment: 4 ½; follow up: 15 | Diagnosis: PTSD  Setting: tertiary centre, out-patients  Country: USA | Completers | N=55, age-adults (M=31.8), all female | CT (n=17)  E (n=14) | 9 | Wait list and supportive counselling | 1. PTSD severity  2. Rape Aftermath Symptom Test  3. Slate-Trait Anxiety Inventory  4. Beck Depression Inventory |
| Foa 1999 | Design: parallel allocation; concealment: unknown; blindness: assessors unaware of treatment condition; attrition: 18% | Treatment: 4 ½ ; follow up: 52 | Diagnosis: PTSD  Setting: tertiary centre, out-patients  Country: USA | Completers and  ITT: last observation carried forward | N=96, age-adults (M= 34.9) all female | Initial allocation unclear. Completers: CT (n=19) E (n=23) | 9 | Wait list and CT+E | 1. PTSD Symptom Scale - Interview  2. Social Adjustment Scale – Global  3. Slate-Trait Anxiety Inventory – State Subscale  4. Beck Depression Inventory |

Note. CT = Cognitive Therapy; E = Exposure; ITT = Intention to Treat; PTSD = Post Traumatic Stress Disorder; M = Mean
