# Supplementary material for: Efficacy of exposure versus cognitive therapy in anxiety disorders: systematic review and meta-analysis
Source: BMC Psychiatry. 2011 Dec 20;11:200. doi: 10.1186/1471-244X-11-200 (PMC3347982; doi:10.1186/1471-244X-11-200)
Supplement: Additional file 4 — Table S3 Studies of cognitive therapy versus exposure in panic disorder with or without agoraphobia. Note. CT = Cognitive Therapy; E = Exposure; ITT = Intention to Treat; PD = Panic Disorder; M = Mean. [file 1471-244X-11-200-S4.DOC]

**Table S3 Studies of Cognitive Therapy versus Exposure in Panic Disorder with or without Agoraphobia**

| **Study** | **Design and study quality** | **Treatment/ follow up (weeks)** | **Participants** | | | | | | |
| --- | --- | --- | --- | --- | --- | --- | --- | --- | --- |
|  |  |  | **Clinical condition and setting** | **ITT** | **Demographic** | **Interventions** | | **Comparison** | **Outcome scales** |
|  | | | | | | **Type** | **No. of sessions** |  |  |
| Bouchard et al 1996 | Design: parallel allocation; concealment: unknown; blindness: unknown;  attrition: 24% | Treatment: 15; follow up: 26 | Diagnosis: Panic with agoraphobia  Setting: University clinic, outpatients  Country: Canada | Completers | N =28 Age – Adults (M= 39) Sex: 14% male | CT (n=14)  E (n=14) | 15 |  | 1. Panic attack diary  2. Agoraphobic Cognition Questionnaire  3. Body Sensation Questionnaire  4. State-Trait Anxiety Inventory  5. Beck Depression Inventory  6. Sheehan Disability Scale |
| Williams and Falbo 1996 | Design: parallel allocation; concealment: unknown; blindness: unknown; attrition: 15% | Treatment: 8; follow up: 104 | Diagnosis: panic with or without agoraphobia  Setting: university clinic, outpatients,  Country: USA | Completers | N= 48 Age 17-73 (M=38) Sex: 16% male | CT (n= 14)  E (n=12) | 8 | CT+E; delayed treatment control | 1.Panic record  2. Self-Efficacy Scales for Agoraphobia  3. Fear Questionnaire  4. Anticipated panic 5. Panic coping self-efficacy  6. Agoraphobic Cognitions Questionnaire  7. Body Sensations Questionnaire  8. Beck Depression Inventory |
| Marchand et al 2008 | Design: parallel allocation; concealment: blind; blindness: assessors blind to treatment allocation; attrition: 11% | Treatment: 18; follow up: 52 | Diagnosis: panic with agoraphobia  Setting: university clinic, outpatients  Country: Canada | Completers | N=154 age18-55 (M=36) Sex: not reported | CT +/-imipramine (n=31)  E +/- imipramine (n=30) | 14 (group) | (CT +E; supportive therapy) +/- imipramine | 1. Agoraphobia Cognitions Questionnaire  2. Body  Sensations Questionnaire  3. Mobility  Inventory for Agoraphobia  4. State–Trait Anxiety  Inventory  5. The Beck Depression Inventory  6. Global Symptom Severity Scale |
| Salkovskis et al 2007 | Design: parallel allocation; concealment: blind; blindness: assessors blind to treatment allocation; attrition: 11% | Treatment: 1 | Diagnosis: panic with agoraphobia  Setting: university clinic, outpatients  Country: UK | Completers | N=16 age: adults (M= 36) Sex: 12% male | CT (n=8)  E (n=8) | 2 | None | 1. Beck Depression Inventory  2. Beck Anxiety Inventory  3. Panic frequency over the previous week  4. Fear Questionnaire  5. Chambless Agoraphobic Cognitions Questionnaire  6. Behavioural walk |
| Arntz et al 2002 | Design: parallel allocation; concealment: unknown; blindness: unknown; attrition: 25% | Treatment: 4; follow up: 26 | Diagnosis: panic without agoraphobia Setting: university clinic, outpatients Country: The Netherlands | Completers and ITT (last observation carried forward) | N=69 Age20-65 (*M* = 34.8), Sex: 61% male | CT (n= 32)  E (n=35) | 12 (mixture of groups and individual) | None | 1. Panic Attack Diary  2. Patient assessed daily  average level of anxiety  rating (0-100)  3. Fear of Fear questionnaire  4. Fear Questionnaire  5. State-Trait Anxiety Inventory  6. Symptoms Check List-90  7. Rating of 1-4 idiosyncratic  assumptions formulated at  first session |
| Clark et al 1994 | parallel allocation; concealment: unknown; blindness: assessors blind to treatment allocation; attrition: 5% | Treatment: 12; follow up: 64 | Panic with or without agoraphobia  Setting: university clinic, outpatients  Country: UK | Partial ITT: attendees of 3 or more sessions | N=64 Age 18-65 (*M* = 34.6), Sex: 22% male | CT (n=20) E (n=20) | 12 (plus 3 boosters) | Imipramine and wait list control | 1. Panic attacks frequency and panic-related distress/disability  2. Beck Anxiety  Inventory  3. Hamilton  Anxiety Rating Scale  4. Body Sensations Interpretation  Questionnaire  5. Fear Questionnaire  6. The Agoraphobic Cognitions Questionnaire  7. Beck  Depression Inventory |
| Arntz and van den Hout 1995 | Parallel allocation; concealment: unknown; blindness: self-report only;  attrition: 3% | Treatment: 12; follow up: 26 | Panic without agoraphobia  Setting: university clinic, outpatients  Country: The Netherlands | Completers and ITT (last observation carried forward) | N=36 Age 21-52 (*M* = 34.1), Sex: 61% male | CT (n=18) E (n=18) | 12 | Wait list (not randomised) | 1. Panic attacks frequency (by panic diary)  2. Fear Questionnaire  3. Fear of Fear questionnaire  4. State-Trait Anxiety  Inventory  5. Depression Symptoms Inventory  8. SCL-90 |

Note. CT = Cognitive Therapy; E = Exposure; ITT = Intention to Treat; PD = Panic Disorder; M = Mean
