# Supplementary material for: Efficacy of exposure versus cognitive therapy in anxiety disorders: systematic review and meta-analysis
Source: BMC Psychiatry. 2011 Dec 20;11:200. doi: 10.1186/1471-244X-11-200 (PMC3347982; doi:10.1186/1471-244X-11-200)
Supplement: Additional file 5 — Table S4 Studies of cognitive therapy versus exposure in social phobia. Note. CT = Cognitive Therapy; E = Exposure; ITT = Intention to Treat. [file 1471-244X-11-200-S5.DOC]

**Table S4 Studies of Cognitive Therapy versus Exposure in Social Phobia**

| **Study** | **Design and study quality** | **Treatment/ follow up (weeks)** | **Participants** | | | | | | |
| --- | --- | --- | --- | --- | --- | --- | --- | --- | --- |
|  |  |  | **Clinical condition and setting** | **ITT** | **Demographic** | **Interventions** | | **Comparison** | **Outcome scales** |
|  | | | | | | **Type** | **No. of sessions** |  |  |
| Hofman 2004 | Design: parallel allocation; concealment: unknown; blindness: unknown; attrition: 18% | Treatment: 12; follow up: 26 | Diagnosis: Social Phobia  Setting: university clinic, outpatients  Country: USA | Completers | N=90 Age: adults (M=28.8 for CT and M= 34.0 for E)  Sex: 54% male | CT (n= 30)  E (n=30) | 12 (group) | Wait list | 1. Social Phobia and Anxiety  Inventory  2. Social Cost Questionnaire  3. Social stress test |
| Clark 2006 | Design: parallel allocation; concealment: unknown blindness: raters unaware of the treatment allocation;  attrition: 5% | Treatment: 14; follow up: 52 | Diagnosis: Social Phobia  Setting: university clinic, outpatients  Country: UK | ITT: (last observation carried forward) | N =62 Age: adults (M= 31.95)  Sex: 56% male | CR (n=21)  E (n=21) | 14 | Wait list | 1. Social Phobia Composite  2. ADIS Fear and Avoidance  3. Social Phobia Scale  4. Social Interaction Anxiety Scale  5. Liebowitz Social Anxiety Scale  6. Social Phobia Weekly Summary Scale  7. Social Phobia Anxiety Inventory  8. Social Phobia subscale  9. Fear of Negative Evaluation  10. Beck Anxiety Inventory  11. Beck Depression Inventory |
| Clark 2003 | Design: parallel allocation; concealment: unknown; blindness: raters unaware of the treatment allocation; attrition: 10% | Treatment 16 | Diagnosis: Social Phobia  Setting: University clinic, outpatients  Country: UK | ITT: (last observation carried forward) | N =60 Age: adults (M= 33.2)  Sex: 48% male | CT (n=20)  E (n=20) | 16 | E + fluoxetine (n=20) | 1. Social Phobia Composite  2. ADIS Fear and Avoidance  3. Social Phobia Scale  4. Social Interaction Anxiety Scale  5. Liebowitz Social Anxiety Scale  6. Social Phobia Weekly Summary Scale  7. Fear Questionnaire  8. Fear of Negative Evaluation  9. Beck Anxiety Inventory  10. Beck Depression Inventory |

Note. CT = Cognitive Therapy; E = Exposure; ITT = Intention to Treat; SP = Social Phobia; M = Mean
